# Value of Radiomics Based on DCE-MRI in distinguishing benign and malignant breast lesions: Predicting Histological Grade and Lymph Node Metastasis of Breast Cancer

**DOI:** 10.12669/pjms.42.1.13121

**Published:** 2026-01

**Authors:** Peiru Li, Hui Xu

**Affiliations:** 1 Peiru Li Department of Radiology, Yongkang First People’s Hospital, Yongkang, Zhejiang Province 321300, P.R. China; 2 Hui Xu Department of Radiology, Yongkang Hospital of Traditional Chinese Medicine, Yongkang, Zhejiang Province 321300, P.R. China

**Keywords:** Breast cancer, DCE-MRI, Differential diagnosis, Histological grade, Radiomics

## Abstract

**Objective:**

The characteristics of benign and malignant breast lesions often overlap and intersect, leading to missed diagnosis or inaccurate diagnosis and excessive biopsy, besides surgical procedures. This study aimed to assess the diagnostic value of radiomics based on dynamic contrast-enhanced magnetic resonance imaging (DCE-MRI) in evaluating the pathological characteristics of breast cancer.

**Methodology:**

This retrospective case-control study included 110 patients with breast lesions who underwent DCE-MRI and obtained pathological results at Yongkang First People’s Hospital and Yongkang Hospital of Traditional Chinese Medicine from September 2019 to December 2024. According to the results, 55 patients with confirmed breast cancer lesions (malignant group) were matched with a cohort of patients with benign lesions (benign group) at a 1:1 ratio. Radiomic parameters from DCE-MRI were analyzed in the two groups.

**Results:**

The radiomic features derived from DCE-MRI showed significant differences not only between benign and malignant breast lesions but also among subgroups stratified by histological grade and axillary lymph node metastasis. Specifically, patients with higher histological grades demonstrated elevated values in long run emphasis and all-angle cluster prominence variance (×10^13^), and decreased values in all-angle correlation, surface-to-volume ratio, and uniformity. Similarly, patients with axillary lymph node metastasis exhibited significantly higher long-run emphasis and cluster prominence variance, and lower uniformity and all-angle correlation (all P < 0.05).

**Conclusion:**

DCE-MRI-based radiomics can effectively differentiate benign and malignant breast lesions and has a predictive value for assessing histological grade and axillary lymph node status. Radiomic features may therefore serve as noninvasive imaging biomarkers to support breast cancer diagnosis, grading, and staging.

## INTRODUCTION

Breast cancer accounts for 7% - 10% of all malignant tumors.^1^,^2^ In recent years, the incidence of breast cancer has been continuously increasing, and the affected population tends to be younger.^2^,^3^ Therefore, implementing individualized and precise treatment for breast cancer is crucial to improve the overall intervention effect of the disease and reduce the risk of death.^3^,^4^ Such an approach requires accurate evaluation of the pathological characteristics of the disease to guide clinical implementation of targeted treatment.^1^-^4^ However, preoperative pathological diagnosis requires an invasive examination method, which may cause complications such as nerve damage and infection.^4^ In addition, this method is associated with false-negative results and cannot reflect the overall nature of the lesion, which limits its clinical application.^5^,^6^

Dynamic contrast-enhanced magnetic resonance imaging (DCE-MRI) and its extension, DCE-MRI radiomics, are an important diagnostic and evaluation method for breast cancer.^7^,^8^ Radiomics uses image information to extract data features, converting medical imaging information into mineable, high-resolution characteristic spatial data, to quantitatively evaluate the internal heterogeneity and morphological characteristics of lesions.^8^,^9^ In recent years, studies have reported that MRI radiomics has an advantageous predictive performance in evaluating benign and malignant breast lesions or axillary lymph node metastasis.^7^-^10^ However, there are few studies using radiomics to comprehensively evaluate benign and malignant breast lesions, different histological grades and the presence or absence of axillary lymph node metastasis.

In recent years, the field of breast cancer radiomics has rapidly evolved, driven by deep learning and multi-omics integration.^11^,^12^ For example, a 2024 multicenter radio-multiomic study demonstrated that combining DCE-MRI features with transcriptomic data significantly improved outcome prediction in breast cancer patients.^11^ Similarly, recent reviews and prospective studies have explored the role of deep-learning–based radiomic signatures in differentiating tumor types and predicting molecular subtypes.^12^–^14^ These developments underscore the importance of updating radiomic approaches to align with clinical decision-making trends.^12^

This retrospective study aimed to analyze DCE-MRI radiomics data from patients with benign and malignant breast lesions, varying histological grades, and with or without axillary lymph node metastasis to determine the diagnostic value of these features.

Compared to previous studies that primarily focused on either lesion differentiation or lymph node evaluation,^7^–^10^ the novelty of this study lies in its comprehensive, three-dimensional approach: we systematically assessed five radiomic features across three major clinical domains (lesion type, histological grade, and nodal metastasis) using a consistently curated dataset. Additionally, the cohort was drawn from two independent clinical institutions over five years, without restriction by molecular subtype or prior treatment, thereby enhancing the real-world applicability of the findings.^11^–^14^ Methodologically, a strict 3D ROI delineation with intraclass correlation coefficient (ICC) analysis was applied to ensure reproducibility, a step often omitted in prior radiomic studies.^9^,^11^–^13^ These strengths offer a more integrated perspective on breast cancer imaging biomarkers.

## METHODOLOGY

Clinical records of 110 patients with breast lesions who underwent DCE-MRI and obtained pathological results at Yongkang First People’s Hospital and the Yongkang Hospital of Traditional Chinese Medicine from September 2019 to December 2024 were retrospectively analyzed.

### Ethical approval:

This retrospective study was approved by the Ethics Committee of Yongkang First People’s Hospital (Approval number: 2025-LW-005; Approval date: March 28, 2025) and the Yongkang Hospital of Traditional Chinese Medicine(Approval number: 2025-003-01; Approval date: March 19, 2025). All patients had previously provided written informed consent at the time of clinical admission and imaging, authorizing the use of their anonymized data for research purposes. All personally identifiable information was de-identified before data analysis, in accordance with institutional and national privacy guidelines.

### Inclusion criteria:

- First discovery of breast lesions
- No prior treatment (surgery, radiotherapy, or chemotherapy) before DCE-MRI examination
- Complete pathological and preoperative imaging data
- Complete imaging and clinical data.

### Exclusion criteria:

- No lesions detected by breast DCE-MRI
- Male patients
- Patients with a history of needle biopsy or neoadjuvant chemotherapy
- Patients with breast implants
- Patients with bilateral breast lesions.

### DCE-MRI examination:

The equipment used included United Imaging 1.436 T and Wandong 1.48 T superconducting whole-body MRI scanning systems and a 10-channel dedicated breast surface coil (Shanghai, China). Patients were instructed to take a prone position with both breasts naturally hanging in the coil. First, a conventional plain scan was performed. Gadodiamide injection (0.1 mmol/kg) was injected through the antecubital vein at a rate of 2.5 ml/s, followed by 13 ml of normal saline at a rate of 2.5 ml/s. Dynamic enhanced scanning was performed 25 seconds after the contrast agent injection. The scanning sequence was an axial three-dimensional small-angle excitation gradient-echo T1WI. Relevant parameters were set as follows: matrix 420×420, field of view 310 mm×310 mm, slice gap 0 mm, slice thickness 2mm, flip angle 15°, TE 2.36 ms, TR 5.2 ms. The duration of a single scan was 58 seconds, with six consecutive scans.

### Image analysis:

One associate chief physician (or one senior attending physician) and one resident physician outlined the region of interest (ROI) using the double-blind method and the intraclass correlation coefficient (ICC) was analyzed. The second scan after contrast agent injection in dynamic enhanced scanning was selected as the research sequence. Using ITK-SNAP Version 3.6 software, ROI was manually outlined layer by layer along the inner edge of breast lesions and suspected metastatic lymph nodes and a three-dimensional image was fused and stored. The original MRI-enhanced images and corresponding ROI three-dimensional segmentations were imported into the Healthcare Analysis Kit software (GE, USA) for radiomic feature extraction. Prior to extraction, all images were preprocessed using a standardized pipeline: images were resampled to isotropic voxel size of 1×1×1 mm³; grayscale intensities were normalized using a fixed bin width of 25; and Gaussian filtering was applied to reduce image noise and variation. Radiomic features were extracted across five major categories: Run - length matrix parameter - Long run emphasis, Texture feature parameter - All - angle cluster prominence variance (×10¹³), Gray - level co - occurrence matrix parameter - All - angle correlation, Morphological parameter - Surface - to - volume ratio, Histogram parameter - Uniformity. Texture features were computed using a 3×3×3 voxel kernel. The reproducibility of feature extraction was ensured by applying the intraclass correlation coefficient (ICC) analysis across two independent readers before final inclusion.

### Statistical analysis:

All analyses were performed using SPSS 25.0 (IBM Corp., Armonk, NY, USA). Data normality was evaluated using the Shapiro-Wilk test. Normally distributed data were expressed as mean ± standard deviation. An independent-samples t-test was used to compare groups. Non-normally distributed data were expressed as median and interquartile range. The Mann-Whitney U test was used for between-group comparisons. Count data were expressed as cases and analyzed by the chi-square test. A P value of < 0.05 was considered statistically significant.

## RESULTS

After matching by age, body mass index (BMI), and menopausal status, well-balanced clinical records of 110 patients with breast lesions were available for comparison of results. Among them, 55 breast cancer patients were matched with a cohort diagnosed with benign lesions at a 1:1 ratio. In the malignant group, the age of patients ranged from 30 to 80 years, with an average of 55.90 ± 11.10 years and the BMI ranged from 17.5 to 26.3 kg/m², with an average of 22.51 ± 2.33 kg/m². There were 16 patients in menopause; 28 cases of left-sided lesions and 27 cases of right-sided lesions. As summarized in Table-I, the pathological types included invasive ductal carcinoma, ductal carcinoma in situ, intraductal papillary carcinoma, lobular carcinoma, and other subtypes. A total of 23 patients had axillary lymph node metastasis. There were no significant differences in the basic characteristics between the groups (P > 0.05).

**Table-I:** Comparison of Basic Characteristics between the Two Groups.

Index	Malignant Group (n = 55)	Benign Group (n = 55)	t/χ^2^	P
Age (years)	55.69±8.63	57.15±9.97	-0.818	0.415
BMI(kg/m²)	22.51±2.33	23.05±2.92	-1.068	0.288
Menopause (Yes)	16 (29.09)	21 (38.18)	1.018	0.313
Left/Right	29/26	26/29	0.327	0.567
***Pathological Type***				
Invasive ductal carcinoma	32 (60.00)	/		
Ductal carcinoma in situ	7 (12.73)	/		
Intraductal papillary carcinoma	5 (9.09)	/		
Lobular carcinoma	7 (10.91)	/		
Other subtypes	4 (7.27)	/		
***Histological Grade***				
I	3 (5.45)			
II	34 (61.82)	/		
III	18 (32.73)	/		
Axillary Lymph Node Metastasis (Yes)	23 (41.82)	/		

The run - length matrix parameter - long run emphasis and the texture feature parameter - all - angle cluster prominence variance (×10¹³) in the malignant group were significantly higher than those in the benign group, while the gray - level co - occurrence matrix parameter - all - angle correlation, the morphological parameter - surface - to - volume ratio and the histogram parameter - uniformity in the malignant group were significantly lower than those in the benign group (all P < 0.05) (Table-II).

**Table-II:** Comparison of DCE - MRI Radiomics Examination Parameters between the Malignant Group and the Benign Group.

Index	Malignant Group (n = 55)	Benign Group (n = 55)	t/Z	P
Run - length matrix parameter - Long run emphasis	1.18±0.09	1.06±0.08	7.549	＜0.001
Texture feature parameter - All - angle cluster prominence variance (×10¹³)	37.81±10.55	19.48±7.74	10.386	＜0.001
Gray - level co - occurrence matrix parameter - All - angle correlation	0.00048±0.00016	0.00069±0.00017	-6.802	＜0.001
Morphological parameter - Surface - to - volume ratio	547(428-632)	854(658-965)	-7.310	＜0.001
Histogram parameter - Uniformity	0.635(0.574-0.694)	0.845(0.758-0.934)	-7.381	＜0.001

As shown in Table-III, patients in the malignant group demonstrated significant differences in the run - length matrix parameter - long run emphasis, the texture feature parameter - all - angle cluster prominence variance (×10¹³), the gray - level co - occurrence matrix parameter - all - angle correlation, the morphological parameter - surface - to - volume ratio and the histogram parameter - uniformity among patients with different histological grades (P < 0.05).

**Table-III:** Comparison of DCE - MRI Radiomics Examination Parameters among Patients with Different Histological Grades in the Malignant Group.

Index	Grade I (n = 3)	Grade II (n = 34)	Grade III (n = 18)	F	P
Run - length matrix parameter - Long run emphasis	1.024±0.153	1.164±0.080	1.230±0.102	7.320	0.002
Texture feature parameter - All - angle cluster prominence variance (×10¹³)	24.21±2.05	36.93±9.80	43.04±11.34	5.152	0.009
Gray - level co - occurrence matrix parameter - All - angle correlation	0.00075±0.00018	0.00053±0.00016	0.00040±0.00014	7.681	0.001
Morphological parameter - Surface - to - volume ratio	712.67±35.13	560.03±112.46	467.72±106.35	8.364	0.001
Histogram parameter - Uniformity	0.840±0.052	0.674±0.102	0.579±0.057	14.224	＜0.001

The run - length matrix parameter - long run emphasis and the texture feature parameter - all - angle cluster prominence variance (×10¹³) in patients with axillary lymph node metastasis were significantly higher than those in patients without metastasis, while the gray - level co - occurrence matrix parameter - all - angle correlation, the morphological parameter - surface - to - volume ratio and the histogram parameter - uniformity in patients with axillary lymph node metastasis were significantly lower than those in patients without metastasis (P < 0.05) (Table-IV).

**Table-IV:** Comparison of DCE - MRI Radiomics Examination Parameters among Patients with Different Axillary Lymph Node Metastasis Statuses in the Malignant Group.

Index	Axillary Lymph Node Metastasis (n = 23)	No Lymph Node Metastasis (n = 32)	t	P
Run - length matrix parameter - Long run emphasis	1.217±0.092	1.152±0.101	2.408	0.020
Texture feature parameter - All - angle cluster prominence variance (×10¹³)	43.98±11.19	34.40±8.99	3.509	0.001
Gray - level co - occurrence matrix parameter - All - angle correlation	0.00042±0.0014	0.00055±0.00018	-2.920	0.005
Morphological parameter - Surface - to - volume ratio	492.23±104.66	568.76±125.21	-2.366	0.022
Histogram parameter - Uniformity	0.604±0.092	0.684±0.106	-2.877	0.006

## DISCUSSION

This study showed that DCE-MRI radiomics parameters can distinguish benign from malignant breast lesions. There was a significant correlation between radiomics parameters and histological grade, as well as between radiomics parameters and the presence or absence of axillary lymph node metastasis.

Accurately predicting the pathological characteristics of breast cancer before treatment is crucial for guiding the implementation of targeted clinical interventions. Among the commonly used imaging examination methods for breast cancer, DCE-MRI has been widely applied and recognized due to its advantages, such as high soft-tissue resolution.^7^-^10^ While previous studies have suggested that enhanced MRI scanning can display the internal heterogeneity and morphology of pathological tissues and lymph nodes, the lack of inter-group consistency in these studies led to differences in the reported diagnostic efficacy.^9^,^10^,^15^,^16^ The radiomics analysis of MRI can quantitatively analyze image information, is objective, and eliminates the influence of human factors.^17^,^18^

The results of this study demonstrated significant differences in the DCE-MRI radiomics parameters between benign and malignant breast lesions, as well as between different histological grades and the presence or absence of axillary lymph node metastasis. These results are consistent with previous studies.^16^-^19^ Furthermore, the results confirm recent advancements reported in high-impact radiomics literature. The role of DCE-MRI–based texture features in predicting tumor aggressiveness has also been demonstrated in multi-institutional studies using deep-learning architectures.^11^ Unlike prior works, this study further explores their association with histological grade and nodal metastasis, thus providing an integrated view of tumor biology from imaging data.

The histogram parameter, uniformity, mainly reflects the uniformity of the distribution of pixels with different gray levels in a grayscale image: a lower value indicates that the differentiation of cancer cells is poor, the degree of heterogeneity of axillary lymph nodes is higher, the possibility of axillary lymph node metastasis is higher, and the growth rate of cancer cells is faster.^20^ The morphological parameter, surface-to-volume ratio, reflects the roundness of the lesion: a smaller value indicates that the shape of the lesion is closer to a circle, indicating more advanced cancer and a higher possibility of axillary lymph node metastasis.^17^ Previous studies have confirmed that the typical shape of axillary metastatic lymph nodes is round rather than reniform.^21^-^23^ The texture feature parameter, all-angle cluster prominence variance, reflects the similarity of different gray-level distributions in the image. A larger value indicates greater dispersion in cluster prominence values and greater heterogeneity of the lesioned tissue.^24^-^25^ The gray-level co-occurrence matrix parameter, all-angle correlation, reflects the symmetry of different gray-level distributions in the image, with a larger value indicating that the pixel gray values in the matrix are more uniform and the heterogeneity is lower.^25^

The run-length matrix parameter, long run emphasis, reflects the spatial positions among pixels in the image. A higher value of this parameter indicates that the image texture is coarser, heterogeneity is greater, and the likelihood of cancer progression and axillary lymph node metastasis is higher.^26^ Thus, it can be seen that the histogram parameter - uniformity, the morphological parameter - surface - to - volume ratio, the texture feature parameter - all - angle cluster prominence variance, the gray - level co - occurrence matrix parameter - all - angle correlation, and the run - length matrix parameter - long run emphasis obtained by the DCE-MRI radiomics method have substantial diagnostic value, as they can identify significant differences between benign and malignant breast lesions, among different histological grades, and between the presence or absence of axillary lymph node metastasis.^20^-^26^

In addition to their diagnostic significance, the radiomic features analyzed in this study may have important clinical implications. For example, an increased value of long-run emphasis, which reflects coarse texture and greater intratumoral heterogeneity, may indicate a higher likelihood of malignancy and could help reduce unnecessary biopsies when considered alongside conventional imaging interpretations. Similarly, features such as low uniformity and decreased all-angle correlation are associated with greater tissue irregularity and may assist in early risk stratification by identifying patients who require expedited surgical planning or further imaging (e.g., PET-CT or targeted ultrasound). As non-invasive imaging biomarkers, these parameters could also be incorporated into artificial intelligence-based clinical decision support systems to improve diagnostic consistency across different institutions, especially in resource-limited settings. These applications highlight the translational potential of radiomics in enhancing precision medicine for breast cancer patients.

One of the key strengths of this study is its comprehensive radiomics framework based on DCE-MRI, which simultaneously addresses three essential aspects of breast cancer imaging evaluation: benign-versus-malignant lesion classification, histological grade differentiation, and axillary lymph node metastasis prediction. Unlike previous studies that often focused on a single outcome, our multidimensional approach provides a more integrated understanding of tumor biology. Additionally, the dataset was sourced from two independent medical institutions using consistent MRI acquisition protocols, which enhances the generalizability and real-world relevance of the findings. The use of a strict manual 3D ROI delineation strategy, coupled with inter-observer reliability assessment, also contributes to the methodological robustness and reproducibility of radiomic feature extraction. Together, these aspects strengthen the scientific validity and clinical translatability of our results.

In interpreting the findings of this study, it is also important to consider the influence of additional potential confounders that were not statistically adjusted for. Variables such as tumor size, breast density, lesion type, and background parenchymal enhancement (BPE) may impact DCE-MRI radiomic parameters in meaningful ways.^15^,^19^,^23^–^25^ For instance, tumor size could alter shape-related metrics such as surface-to-volume ratio;^19^,^25^ BPE and breast density may affect enhancement kinetics, altering histogram-based and texture-derived features;^15^,^23^ and lesion type heterogeneity might lead to variability in spatial texture patterns.^24^–^26^ While age, BMI, and menopausal status were matched between groups, these other variables were not included as covariates due to sample size constraints. Therefore, caution should be exercised when generalizing radiomic differences solely based on group comparisons.

### Limitations

First, it was a dual-center retrospective analysis with a relatively small sample size, which may limit the generalizability of the findings. Second, although consistency checks were performed, variability in ROI delineation may still exist due to differences in operator experience. Third, some potential confounding variables—such as tumor size, breast density, lesion type, and background parenchymal enhancement (BPE)—were not included as covariates in the statistical models, potentially introducing residual bias. Furthermore, subgroup analyses based on age, menopausal status, or tumor size were not performed due to the limited sample size and the matched case–control design. Conducting such analyses would compromise statistical power, violate the matching structure, and increase the risk of type I error. Future prospective multicenter studies with larger, more diverse cohorts are warranted to incorporate these variables into multivariable frameworks and to validate the generalizability of radiomic features across subpopulations.

## CONCLUSION

Radiomic parameters from DCE-MRI can be used to identify benign and malignant breast lesions, assess histological grade, and evaluate axillary lymph node metastasis. The results of this study suggest that the method may be used for guiding the implementation of targeted clinical treatment.

### Recommendations

Future studies should develop a radiomics prediction model to better identify patients with benign and malignant breast lesions, different histological grades, and the presence or absence of axillary lymph node metastasis.

### Authors’ contributions:

**PL:** Study design, literature search and manuscript writing.

**PL and HX:** Data collection, data analysis and interpretation. Critical review.

**PL:** Manuscript revision and validation and is responsible for the integrity of the study.

All authors have read and approved the final manuscript.
